# Identifying Root-Associated Endophytic Fungi and Bacteria in *Festuca* and *Lolium* Grasses from a Site in Lithuania

**DOI:** 10.3390/microorganisms13040799

**Published:** 2025-03-31

**Authors:** Violeta Stakelienė, Izolda Pašakinskienė, Saulė Matijošiūtė, Justas Martūnas, Gitana Štukėnienė

**Affiliations:** 1 Botanical Garden, Vilnius University, Kairėnų 43, 10239 Vilnius, Lithuania; izolda.pasakinskiene@gf.vu.lt (I.P.);; 2 Life Sciences Centre, Vilnius University, Saulėtekio 7, 10221 Vilnius, Lithuania

**Keywords:** Poaceae grasses, Ascomycota, Basidiomycota, *Bacillus*, growth-promoting fungi and bacteria

## Abstract

This study investigates the diversity and distribution of root endophyte fungi and bacteria across *Festuca* and *Lolium* grasses, including open-grassland and forest species. The species examined include perennials such as *Festuca arundinacea*, *F*. *gigantea*, *F*. *pratensis*, *Lolium perenne*, and *L*. *perenne* × *F*. *gigantea* hybrids and the annuals *L*. *temulentum* and *L*. *multiflorum*. A total of 21 fungal species (60 isolates) and 26 bacterial taxa (59 isolates) were recovered in the culture (PDA medium for fungi and LB for bacteria) from the root cuttings of these grasses. *Microdochium bolleyi* fungi and *Bacillus* sp. bacteria were the most prevalent endophytes, with each being identified in five of the seven plant species examined. The annuals *L*. *multiflorum* and *L*. *temulentum* exhibited a higher abundance of endophytes than that in their perennial relatives, suggesting the benefits of microbial associations in supporting their short life cycles. The woodland *F*. *gigantea* demonstrated the highest fungal endophyte diversity, with six species identified. In contrast, the open-grassland perennials *F*. *arundinacea*, *F*. *pratensis*, and *L*. *perenne* hosted only one to two species. Two Basidiomycota, *Coprinellus disseminatus* and *Sistotrema brinkmannii*, were exclusively obtained from the roots of the forest grass *F*. *gigantea*. Notably, the open-grassland perennial *F*. *arundinacea* exhibited the highest bacterial diversity, with nine species present. However, it showed the lowest fungal diversity, with only one species detected. Overall, our study reveals distinct patterns of fungal and bacterial endophyte diversity in the roots of *Festuca* and *Lolium* grasses, with variations linked to host species, growth type traits, and ecological adaptations. Among the root-derived endophytes isolated, several fungi and bacteria are potential candidates for plant growth promotion and biocontrol. Therefore, the findings of this study provide potential implications for improved grassland management and crop breeding strategies aimed at specific climate and/or soil conditions.

## 1. Introduction

Endophytic microorganisms comprising fungi and bacteria are ubiquitous in plants, colonizing the above-ground parts and roots of the plants without causing apparent harm. Endophytic fungi are common in meadows, the Alps, deserts, and forests of the middle zones [1,2,3,4,5,6]. Many well-known endophytes of the anamorphic Ascomycota phylum are characterized by melanized septate hyphae. Due to this feature, these fungi are assigned into a special group of dark septate endophytes, DSEs [7,8]. These endophytic microorganisms can provide nutritional and protective benefits to their hosts. Numerous endophytic fungi have a beneficial impact on plants, helping them to resist biotic and abiotic stress, fighting against pests, and promoting their growth and development [5,9,10]. Just like fungi, diverse bacteria have been shown to promote plant growth and have been assigned as plant-growth-promoting rhizobacteria or plant-growth-promoting bacteria [11,12].

Numerous studies indicate that the majority of grasses are hosts to endophytic fungi [4,13,14,15]. In this respect, *Festuca*/*Lolium* (Poaceae) plants have been extensively studied for the *Epichloë*/*Neotyphodium* endophyte associations in their foliar parts and seeds [16,17,18,19,20,21,22,23,24]. These fungi systemically colonize the leaves and stems of host plants but not the roots. In contrast to the wide research on *Epichloë*/*Neotyphodium* in the above-ground parts, there is only limited knowledge of the root-associated endophytes in *Festuca*/*Lolium* grasses so far, which are cultivated worldwide in pastures and meadows, providing nutrition for livestock. Up until now, the fungal and bacterial endophytes in the roots of *Festuca* spp. from the Schedonorus group have only been described for *Festuca gigantea* [25], while for *F. arundinacea*, specifically, root-derived bacterial endophytes have been studied [26]. In the foliar parts, an analysis conducted by Przemieniecki et al. [27] revealed specific patterns of fungal endophytes colonizing the forage grasses *Lolium perenne* and *Phleum pratense*.

A limited number of studies has examined fungal and bacterial endophytes within the same experiment, either in relation to a specific plant or an ecosystem [15,25,28,29,30]. Toju and co-authors [30] examined more than 100 grassland plant species and showed that a plant’s below-ground microbiome has different features and dynamics compared to those in the above-ground microbiome. The root system is essential for anchoring the plant in the soil and for absorbing the water and minerals necessary for its growth and survival. Additionally, studies have shown that the roots play a critical role in sensing environmental factors. They act as a sensory hub, influencing plant growth and morphogenesis in response to changes in its surroundings [31,32]. Given that the roots host a diverse range of bacteria and fungi, research into the complexity of the microbial communities in these hidden parts of plants is crucial for advancing our understanding of plant life.

This study aims to fill in a knowledge gap by assessing the diversity and prevalence of the fungal and bacterial communities in the roots of grasses through an isolated culture approach. Overall, its results provide insights into the endophyte distribution patterns across *Festuca* and *Lolium* species, which show contrasts based on their ecological habitats and seasonal growth characteristics. These findings contribute to a deeper understanding of plant–microbe interactions in agricultural and ecological contexts.

## 2. Materials and Methods

### 2.1. Root Sampling and Sterilization

The plants were grown from young tiller transplants grown in the experimental field of the Botanical Garden of Vilnius University (Vilnius, Lithuania; 54.7362067° N, 25.403482° E). The species examined included the perennials *F*. *arundinacea*, *F*. *gigantea*, *F*. *pratensis*, *L*. *perenne*, and *L*. *perenne* × *F*. *gigantea* hybrids and the annuals *L*. *temulentum* and *L*. *multiflorum*. *F*. *gigantea* is a woodland species, whereas all of the other species are grasses from open-grassland sites used in meadows and pastures, except for *L*. *temulentum*, which is a short-lived weed (Table 1). Plant tillers were collected from the experimental field in May–June. In each species, root samples were obtained from 25–30 plants, with each plant represented by three tillers. For preparation of the root samples, the collected tillers were washed under running tap water, and the old roots were removed. Tillers with no roots were placed in test tubes with added tap water. New healthy roots, 1–2 cm long, were collected and surface-sterilized accordingly using 50% ethanol for 90 s and 1.25% sodium hypochlorite for 90 s; after this, the samples were washed 3 times for 3 min with sterile water. In addition, 200 μL of final wash water was added to three Petri dishes with the Potato Dextrose Agar (PDA) or Luria Broth (Miller’s LB broth) (LB) medium during the fungal and bacterial culture from the roots. This was used as a negative control to confirm that the root sterilization was adequate.

### 2.2. Microscopic Evaluation and Estimation of the Abundance of Endophytic Fungi in the Grass Roots

For the microscopic evaluation, newly grown roots, as described in Section 2.1 (excluding the sterilization step), were used. Root segments were collected and placed in 1.5 mL Eppendorf test tubes with a fixative of ethanol–glacial acetic acid (3:1) and kept in a refrigerator (at 2–3 °C) until their further use. Prior to microscopy, all of the roots were softened using the following enzyme treatment: the sampled roots were washed twice with citrate buffer (0.1 M, pH 4.8) at 27–28 °C for 10 min and treated with 0.5% Macerozyme R-10 at 37 °C for 25 min.

After the enzyme treatment, the roots were washed with citric citrate buffer and stained with 0.025% Trypan Blue, following the protocol described by Kiheri [33] with some modifications: the roots were stained at 90 °C for 30 min and then bleached with a lactic acid–glycerol (1:1) mixture, repeating this three times. The first two bleaches took place at 37 °C for 30 min, while during the third, they were left at room temperature for 24 h before the microscopic analysis. Lactic acid–glycerol (4:1) was used to prepare the root tip sections on the microscopy slides.

To determine the frequency of fungal endophyte colonization and compare it between species, meristem tip cuttings from *N* = 120 roots were analyzed microscopically for each species. The presence of fungal structures such as septate and melanized hyphae and agglomerates of fungal spores, as specified in [25], were assessed using a Nikon ECLICE Ci-L phase contrast microscope (Nikon, Tokyo, Japan) in 10 fields of view for each root at ×400 magnification.

### 2.3. Isolation of the Fungi

To make the PDA medium, 200 g of peeled, sliced potatoes (Lithuanian var. Rasa) was boiled in 1 L of distilled water for 30 min. The potato mass was filtered through a cheesecloth, saving the effluent. The potato infusion was poured into flasks 200 mL at a time. A total of 4 g of dextrose and 4 g of agar were added into each flask. The mixtures were autoclaved at 121 °C for 20 min. Ampicillin sodium salt (final concentration—100 mg/L) and streptomycin sulfate (final concentration—100 mg/L) were added prior to distribution of the agar into the plates to inhibit the bacterial growth selectively.

For cultivation of the fungal endophytes, *N* = 200–250 root tip fragments per species were used; these samples were collected from 25–30 plants. Five cuttings of the surface-sterilized roots were placed into each Petri dish with the PDA medium. Before isolation, the roots were squashed using a sterile needle to facilitate the proliferation of endophytic fungi. The samples were incubated in the dark at 27 °C. After 7–14 days, we observed the growth of fungal colonies in proximity to the root segments. The fungal isolates obtained were deposited into the laboratory collection of Vilnius University Botanical Garden.

### 2.4. Isolation of the Bacteria

A total of 25 g of LB powder (Thermo Fisher Scientific Baltics, Vilnius, Lithuania) was dissolved in 1 L of purified water, heated and agitated until it completely dissolved, and then sterilized through autoclaving at 121 °C for 15 min. The surface-sterilized roots were prepared as described in Section 2.1. For cultivation of the bacterial endophytes, *N* = 100 root tip fragments per species were used; these samples were collected from 25–30 plants. Root cuttings were incubated in Petri dishes with the LB medium at 37 °C in the dark. After 1–2 days, we observed the bacterial colonies in proximity to the root segments.

### 2.5. DNA Extraction from the Fungi and Bacteria

For the extraction of genomic DNA from the fungi, 10-day-old fungal colonies grown on the PDA medium were used, sampling 100 mg of mycelial biomass. For bacteria, 1- to 2-day-old colonies were sampled and transferred into the liquid LB medium for growth. The next day, genomic DNA was extracted from these cultures.

The fungal and bacterial genomic DNA was isolated using the Quick-DNA^TM^ HMW MagBead Kit (Zymo Research, Irvine, CA, USA), following the manufacturer’s guidelines.

### 2.6. Standard DNA Amplification and Sequencing

For standard DNA amplification, the primer pairs used in the PCR reactions are listed in Table 2 for fungi and bacteria, respectively. The total volume of the PCR mix for amplification was 50 μL. PCR was conducted under a temperature profile of 94 °C for 3 min, followed by 35 cycles of 94 °C for 30 s, 49–61 °C [calculated according to the primer’s Ta = Tm − (0–4 °C)] for 30 s, and 72 °C for 1 min and a final extension at 72 °C for 5 min.

The PCR products were purified using the GeneJET PCR Purification Kit (Thermo Fisher Scientific Baltics, Vilnius, Lithuania). The sequencing was performed by BaseClear B.V., Leiden, the Netherlands. The sequences from the fungal and bacterial isolates were analyzed against NCBI reference data using the BLAST tool (version 2.16.0) at https://blast.ncbi.nlm.nih.gov/Blast.cgi (accessed on 26 August 2024).

### 2.7. Morphological Characterization of the Endophytic Fungi

The fungal isolates were also characterized according to their morphological features, including their structure, color, and colony edge. A mixture of lactic acid–glycerol (4:1) was used to analyze and photograph the fungal mycelium in each specimen. A microscopic Nikon ECLICE Ci-L phase-contrast microscope was used for viewing.

### 2.8. Statistical Analysis

Isolation frequency (IF): The percentage of the number of endophytic fungal and bacterial strains isolated out of the total number of isolates was obtained. This parameter was used to determine the dominance of the endophytic species accessed.

Margalef’s index D = (S − 1)/lnN was used to analyze taxon richness. S here is the total number of taxa, while N is the total number of isolates. These parameters were calculated by applying the formula used by Song et al. [37].

The data were analyzed using a one-way ANOVA in STATISTICA^®^ 7.0. The statistical significance of the differences between the means was assessed using a post hoc Tukey’s test. Differences were considered to be significant at *p* ≤ 0.05. Charts were drawn using MS Excel software 2016.

### 2.9. Photography

Images of the colonies were taken using a Sony Alpha a6300 camera (Sony Corporation, Tokyo, Japan) with a Sigma 56 mm f/1.4 lens (Sony Corporation, Tokyo, Japan). Root section cuttings and mycelium samples were analyzed under the phase-contrast microscope, and the NIS-Elements D software (version 6.02.01) program was used for the microscopic photography and analysis.

## 3. Results

### 3.1. The Frequency of Colonization by Endophytic Fungi in Festuca and Lolium Species and Their Hybrids

**Figure 1:**
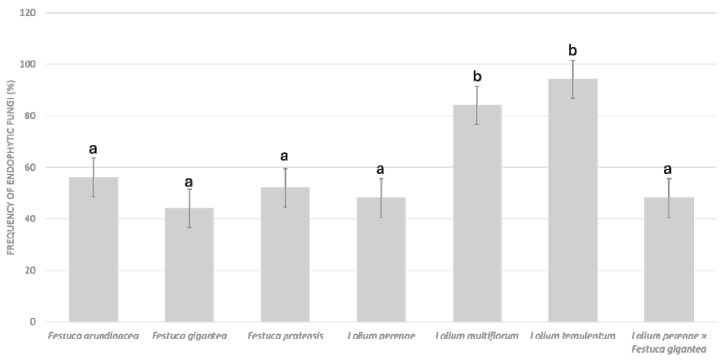
Colonization frequency of endophytic fungi in root tip cuttings of *Lolium* and *Festuca* species and *L*. *perenne* × *F*. *gigantea* hybrids. Different letters above the bars indicate significant differences between the species (*p* ≤ 0.05) based on Tukey’s HSD post hoc test.

In the *Festuca* species and the hybrids, fungal structures were observed in 44% to 56% of the roots examined (Figure 1). The highest prevalence of endophytic fungal structures was observed in the roots of the annuals *L*. *temulentum* (94%) and *L*. *multiflorum* (84%) in contrast to the perennial *L*. *perenne* (48%). The colonization frequency in the *L*. *perenne* × *F*. *gigantea* hybrids was at the level observed in their parental species.

### 3.2. Diversity and Abundance of the Endophytic Fungi

**Figure 2:**
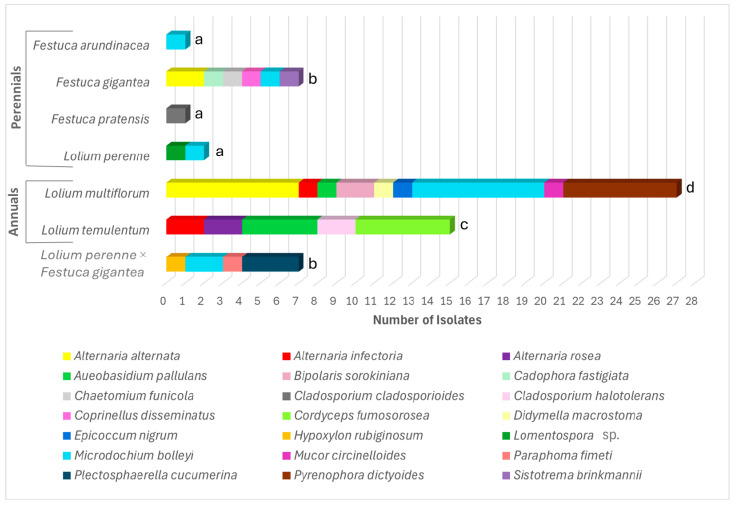
Distribution of endophytic fungi isolated from the roots of *Festuca* and *Lolium* species and their hybrids. All of the species are classified under Ascomycota, except for *Coprinellus disseminatus* and *Sistotrema brinkmannii* from Basidiomycota and *Mucor circinelloides* from Mucoromycota. Different letters above the bars indicate significant differences between the species (*p* ≤ 0.05) based on Tukey’s HSD post hoc test. Note: The laboratory-produced *L*. *perenne* × *F*. *gigantea* hybrids are perennial-type plants.

The fungal endophyte distribution across the species examined is presented in Figure 2. Endophytic fungi were isolated from surface-sterilized fresh root cuttings placed on the PDA medium, sampling *N* = 200–250 for each of the seven species. No colony growth was detected in the control Petri dishes with the final root wash water after sterilization. The taxonomic assignment was based on the colony morphology and the cytomorphological characteristics of the species, which are shown in Figure S1. In addition, taxonomy was confirmed through the alignment of the PCR-produced ITS *RPB2*, *SSU*, and *TEF1*-*a* sequences with reference fungal DNA data, following the directions in Vu et al., 2019 [38] (Table S1). It should be noted that our study used previously published data on fungal endophytes in *F*. *gigantea*, as described by Pašakinskienė et al. [25]. Detailed descriptions of the fungal morphotypes of the six species detected in *F*. *gigantea* can be also found in the paper [25].

The pattern of the fungal endophyte distribution in the roots of the *Festuca* and *Lolium* species and their hybrids is shown in Figure 2. A total of 60 fungal isolates, representing 21 species, were obtained from the roots of these grasses. Among these, 18 species were identified as belonging to the phylum Ascomycota—*Alternaria alternata*, *Alternaria infectoria*, *Alternaria rosea*, *Aureobasidium pallulans*, *Bipolaris sorokiniana*, *Cadophora fastigiata*, *Chaetomium funicola*, *Cladosporium cladosporioides*, *Cladosporium halotolerans*, *Cordyceps fumosorosea*, *Didymella macrostoma*, *Epicoccum nigrum*, *Hypoxylon rubiginosum*, the *Lomentospora* sp., *Microdochium bolleyi*, *Paraphoma fimeti*, *Plectosphaerella cucumerina*, and *Pyrenophora dictyoides*—while 2 Basidiomycota species, *Coprinellus disseminates* and *Sistotrema brinkmanii*, and *Mucor circinelloides* from Mucoromycota were also found (Figure 2 and Figure S1, Table S1).

From Basidiomycota, *Coprinellus disseminatus* and *Sistotrema brinkmannii* were found in the forest grass *F*. *gigantea* (Figure 2), and *Mucor circinelloides* from Mucoromycota was detected in *L*. *multiflorum*.

Among the *Lolium* species examined, the annual *L*. *multiflorum* exhibited the highest fungal diversity, with nine distinct fungal taxa isolated from its roots (Figure 2). Within the *Festuca* group, the woodland grass *F*. *gigantea* showed the greatest fungal diversity, with six fungal endophyte species recovered. A similar rate of fungal diversity by species number was observed in *L*. *temulentum* and the *L*. *perenne* × *F*. *gigantea* hybrids, with five and four taxa, respectively. The lowest diversity was recorded in the open-grassland perennials, namely *L*. *perenne*, *F*. *arundinacea*, and *F*. *pratensis*. From these plants, only one or two endophytic fungal species were recovered, with just one or two isolates obtained (Figure 2). No correlation was found for the presence of fungal taxa when comparing the root endophytes of the parental species *L*. *perenne* and *F*. *gigantea* with those of their *L*. *perenne* × *F*. *gigantea* hybrids, except for *M*. *bolleyi*, which was obtained in both the parents and the hybrids (Figure 2).

As for the isolation frequency (IF, %), the species of the endophytic fungi are presented hierarchically in Table S2. The most prevalent species was *M*. *bolleyi*. It was exceptionally common across the species examined, yielding 12 isolates (IF = 20%), and was found to colonize the roots in five species, except *F*. *pratensis* and *L*. *temulentum* (Figure 2). Another widespread species was *A*. *alternata,* with nine isolates recovered (IF = 15%) (Figure 2). Both *M*. *bolleyi* and *A*. *alternata* reached the highest number of seven isolates from *L*. *multiflorum* as a host species, with each at an IF = 12% (Figure 2). The highest number of 27 isolates was obtained from the roots of *L*. *multiflorum*, followed by *L*. *temulentum* with 15 isolates, 45% and 25% of the total, respectively (Figure 2). This aligns with the high frequency of fungal colonization observed microscopically in the roots of these two annual species (Figure 1). The woodland grass *F*. *gigantea* and the *L*. *perenne* × *F*. *gigantea* hybrids each yielded seven isolates. In contrast, the open-grassland perennials (*F*. *pratensis*, *F*. *arundinacea*, and *L*. *perenne*) had the lowest frequency, with only one or two isolates obtained per species.

### 3.3. Diversity and Abundance of the Endophytic Bacteria

**Figure 3:**
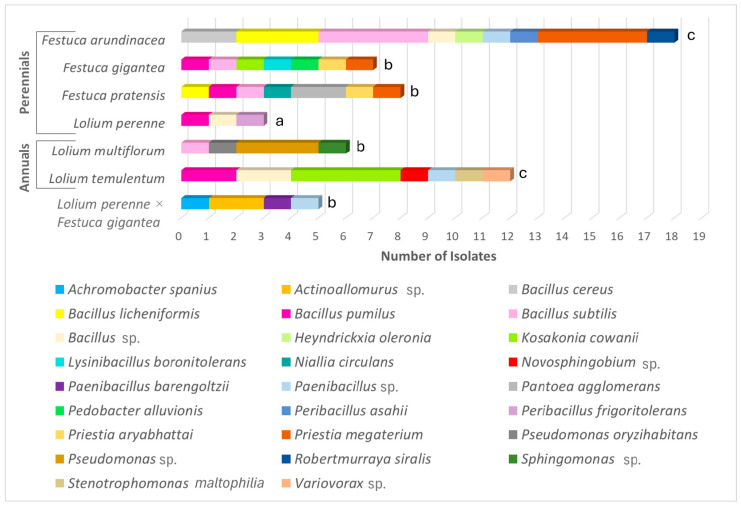
Distribution of endophytic bacteria isolated from *Lolium* and *Festuca* species and their hybrids. Different letters above the bars indicate significant differences between the species (*p* ≤ 0.05) based on Tukey’s HSD post hoc test. Note: The laboratory-produced *L*. *perenne* × *F*. *gigantea* hybrids are perennial-type plants.

The pattern of the endophytic bacterial distribution in the roots of the *Lolium* and *Festuca* species and their hybrids is shown in Figure 3. Twenty-six bacteria taxa were identified from a total of 59 isolates in the culture on the LB medium from the roots of *Festuca* and *Lolium* species and the *L*. *perenne* × *F*. *gigantea* hybrids. No bacterial growth was detected in the control Petri dishes with the final root wash water after sterilization. The taxonomic assignment of the bacteria was confirmed by the BLAST results for the 16S rDNA sequences (Table S3). The 16S rDNA sequences obtained from our bacterial isolates were deposited into GenBank (Table S4).

Notably, the open-grassland perennial *F*. *arundinacea* stood out with the highest bacterial endophyte diversity, yielding 18 isolates (30% of the total) from nine bacteria species (Figure 3, Table S3). The next species with the highest bacterial endophyte diversity was the annual *L*. *temulentum*, with 12 (20%) isolates from seven bacterial taxa. From the roots of *F*. *gigantea* and *F*. *pratensis*, seven bacterial taxa were obtained from each species, yielding seven (12%) and eight (14%) isolates, respectively. *L*. *multiflorum* and *L*. *perenne* × *F*. *gigantea* yielded four bacterial taxa each. In contrast, *L*. *perenne* showed the lowest bacterial associations, with only three endophytic bacterial taxa identified, each represented by a single culture strain.

Similar to the fungal endophyte distribution (Figure 2), the presence of bacterial taxa showed no correlation between the root-derived bacteria of the *L*. *perenne* and *F*. *gigantea* parents and those of the *L*. *perenne* × *F*. *gigantea* hybrids (Figure 3).

As for the isolation frequency (IF, %), the genera of the endophytic bacteria are presented hierarchically in Table S2. The most common were Gram-positive, endospore-forming bacteria from Bacillota (synonym Firmicutes); they were represented by 15 species belonging to eight genera, namely the *Bacillus*, *Heyndrickxia*, *Lysinibacillus*, *Niallia*, *Paenibacillus*, *Peribacillus*, *Priestia*, and *Robertmurraya* genera (Figure 3). Of these, *Bacillus* (IF = 37%), *Priestia* (IF = 14%), and *Paenibacillus* (IF = 7%) were the most prevalent (Figure 3). The set of 15 Bacillota species was identified as follows: *Bacillus cereus*, *Bacillus licheniformis*, *Bacillus pumilus*, *Bacillus subtilis*, the *Bacillus* sp., *Heyndrickxia oleronius*, *Lysinibacillus boronitolerans*, *Niallia circulans*, *Paenibacillus barengoltzii*, the *Paenibacillus* sp., *Peribaccilus asahii*, *Peribaccilus frigoritolerans*, *Priestia aryabhattai*, *Priestia megaterium*, and *Robertmurraya siralis*.

In addition, nine species of Gram-negative bacteria belonging to the Pseudomonadota phylum were isolated and identified as follows: *Achronobacter spanius*, *Kosakonia cowanii*, the *Novosphingobium* sp., *Pantoea agglomerans*, *Pseudomonas oryzihabitans*, *Pseudomonas* sp., the *Sphingomonas* sp., *Stenotrophomonas maltophilia*, and the *Variovorax* sp. (Table S2, Figure 3). Of these, the *K*. *cowanii* and *Pseudomonas* sp. isolates were recovered at higher rates than a single isolate, with an IF = 8% and an IF = 7%, respectively (Figure 3).

The bacteria from the Actinomycetota and Bacteroidota phyla were rare taxa associated with the studied grasses, with each represented by a single species: *Actinoallomurus* sp. (Actinomycetales) was obtained from the hybrid (two isolates) and *Pedobacter alluvionis* (Sphingobacteriales) from *F*. *gigantea* (a single isolate), respectively (Figure 3, Table S2).

### 3.4. The Fungal and Bacterial Taxon Richness Across Plant Species

We calculated the endophyte abundance and taxon richness (D) for the endophytic fungi and bacteria in different plant hosts. Notably, the woodland grass *F*. *gigantea* had the highest microbial endophyte richness, namely D = 2.57 for fungi and D = 3.08 for bacteria (Table 3). The next species with the highest fungal endophyte richness was *Lolium multiflorum* at D = 2.43. For the bacterial associations, all three *Festuca* species—*F*. *arundinacea*, *F*. *gigantea*, and *F*. *pratensis*—and *L*. *temulentum* exhibited high bacterial endophyte richness. The bacterial diversity indices (D) for these species were >2, in contrast to the other host plants, which had diversity indices <2 (Table 3). Perennial polyploid *Festuca* species of natural hybrid origin, such as *F*. *arundinacea* and *F*. *gigantea*, exhibited a significantly higher total abundance of microbial endophytes. In contrast, the related diploid species like *F*. *pratensis*, and particularly *L*. *perenne*, had the lowest abundance (Table 3).

## 4. Discussion

Our findings highlight significant variations in the colonization frequency, taxonomic diversity, and richness of endophytic fungi and bacteria among *Festuca* and *Lolium* grasses, offering insights into the factors that shape the endophyte communities in their roots.

### 4.1. The Distribution of Fungal Root-Derived Endophytes in Festuca/Lolium Grasses

Numerous studies have demonstrated that the roots of most grasses serve as habitats for endophytic fungi [4,13,14,15]. Research on agricultural crops, including wheat, barley, soybean, corn, rice, and cotton, has also revealed a diverse range of fungal endophytes and their essential roles in supporting host plants [1,2].

In the root cutting culture on the PDA medium, a total of 21 fungal species (60 isolates) were isolated, predominantly from the division Ascomycota (18 species), with a few representatives from Basidiomycota (2) and Mucoromycota (1). This aligns with previous studies showing the dominance of Ascomycota among fungal endophytes, which are common root-associated symbionts and saprotrophs [6,27,30,39,40].

Most of these root fungi are known to be saprophytic or endophytic, and they are found in the soil as decomposers of organic matter or internally in various plant parts. However, some of them are also plant pathogens, namely *A*. *alternata*, *A*. *infectoria*, *B*. *sorokiniana*, *P*. *cucumerina*, and *P*. *dictyoides*; they cause diseases in cereals and some other crops. Additionally, they can live endophytically, having a neutral effect on plant health.

The most prevalent root endophyte was *M*. *bolleyi*, found in five of seven host species at an IF = 20% of the total isolate number. Another widely detected fungus was *A*. *alternata* (IF = 15%); it was particularly abundant in *L*. *multiflorum*, with the highest number of isolates (seven) recovered. Both *M*. *bolleyi* [27,41,42,43] and *A*. *alternata* [27,40,44,45] are referred to as plant endophyte generalists and are widely common across diverse plant species.

Along with the grass host generalists, some unusual accessions were isolated from the roots of *Festuca*/*Lolium*. Two Basidiomycota, *Coprinellus disseminatus* (Agaricales) and *Sistotrema brinkmannii* (Cantharellales), were occasionally present in the forest grass *F*. *gigantea*. While *C*. *disseminatus* is not typically an endophyte, it has been found in association with orchids in tropical forests [46,47]. In grasses, this endophytic fungus was obtained in a culture of the leaves of timothy (*Phleum pratense*) grass [27]. *S*. *brinkmannii* is not a typical grass endophyte either; it is a wood-rotting fungus widespread on the bark of trees and also found in the soil [48,49]. However, considering the wide distribution of *C*. *disseminatus* and *S*. *brinkmannii* in terrestrial habitats [49], it can be expected that these endophytes may also be found in the roots of forest grasses.

*Cordyceps fumosorosea* (Hypocreales) was occasionally present in the annual weed *L*. *temulentum* at a high rate (five isolates) of recovery. *Cordyceps* species are known as parasites that inhabit insect larvae [50,51]. While *Cordyceps* fungi are best known for their role as endoparasites and entomopathogens, some species have been isolated from plant tissues as well. A list of *Cordyceps* fungi that naturally infect plants is available in Vega’s review [52]. Notably, in endophyte research, the horizontal transmission of endophytic fungi between insects and plants is a well-known phenomenon, as reviewed by Raman and Suryanarayanan [53]. Given that *L*. *temulentum* (a common weed) thrives in a wide range of disturbed ecological sites, such as waste grounds rich in organic decay which attract numerous insects and their larvae, the presence of *C*. *fumosorosea* in these environments seems highly possible. Overall, plant endophytes can be transmitted either vertically (directly from the parent via seeds) or horizontally (from the surrounding environment) [54,55]. Further detailed studies are needed, particularly on seed-borne endophyte associations, to clarify the origin of the endophytes found in the roots of *Festuca* and *Lolium* plants.

From the fungal endophytes obtained in this study, three root-derived fungi have already been studied for their growth-promoting effects in *L*. *multiflorum* and barley (*Hordeum vulgare*) [10]. In this study, *C*. *fastigiata*, *P*. *fimeti*, and *P*. *cucumerina* promoted the growth of barley and ryegrass, with the most pronounced impact on their root size. In addition, the VOCs (Volatile Organic Compounds) emitted by these fungi exhibited a strong stimulating effect on root growth [10]. Several other fungal endophytes among those obtained have also been identified as growth-promoters. Namely, *A*. *pullulans* [56], *C*. *cladosporioides* [57], and *E*. *nigrum* [58] have demonstrated the ability to produce growth-promoting substances and act as biocontrol agents, helping plants resist pathogens and abiotic stresses.

### 4.2. Fungal Endophytes Across Grass Growth Types and Habitats

Most grasses are home to endophytic fungi, which play an important role in the ecophysiology of plants. Endophytic fungi are particularly prevalent in high-stress environments and are abundant across various ecosystems [4,13,14,15]. The *Festuca*/*Lolium* group examined in our study consists of plants from contrasting environments and of different growth types. *Festuca gigantea* is a woodland species, whereas all of the other species are grasses from open-grassland sites used in meadows and pastures, except for *L*. *temulentum*, which is a short-lived weed. In this tested group of grasses, *F*. *gigantea* stays notably distinct from the others—it has adapted to deal with light deficiency in a specific ecological niche rich in decomposing organic litter shaded by the tree canopy [59].

In this study, when comparing the endophyte prevalence across different plant species, the annual *L*. *multiflorum* exhibited the highest fungal diversity, with nine distinct fungal taxa isolated from its roots. In terms of the isolates recovered, both annual *Lolium* species, *L*. *multiflorum* and *L*. *temulentum*, exhibited the best results. The higher fungal prevalence in the annuals shows the possible benefits of endophytes to these fast-growing *Lolium* species. High levels of endophytic fungi in the roots are likely to have a positive impact on their nutrient acquisition, supporting the short-season growth dynamics of these plants.

Among the perennials, the greatest fungal diversity was recorded in the roots of the woodland grass *F*. *gigantea*. In contrast, the open-grassland perennials *F*. *pratensis*, *F*. *arundinacea*, and *L*. *perenne* exhibited the lowest fungal diversity. This pattern indicates that habitat type plays an important role in shaping fungal endophyte communities, highlighting that forest sites provide more favorable conditions for hosting diverse endophytic fungi than open grasslands.

### 4.3. The Distribution of Bacterial Root-Derived Endophytes in Festuca/Lolium Grasses

Endophytic bacteria are known for their plant-growth-promoting properties, contribution to plant development, and biocontrol effects, similar to endophytic fungi [15,60]. However, the diversity and functional roles of the root-associated bacteria in *Festuca* and *Lolium* species are poorly documented. Among the species examined, *F*. *arundinacea* and *F*. *gigantea* remain the only species studied in terms of the bacterial endophytes derived from their roots [26,27].

In our study, we identified 26 bacterial species from 59 isolates representing members of four bacterial phyla. The most common were Gram-positive, spore-forming bacteria from the Bacillota (syn. Firmicutes) phylum, yielding 58% of the isolated bacterial cultures.

*Bacillus* bacteria were the most prevalent, comprising five species and accounting for 38% of the total isolates. *Bacillus* endophytes were found to be hosted by all of the *Lolium* and *Festuca* species examined, except for the hybrids. *Bacillus* bacteria are widely distributed in natural environments and exhibit remarkable host diversity. These microorganisms perform a broad range of ecological functions, including the decomposition of organic matter, the promotion of plant growth, and the suppression of pathogenic organisms [61,62]. In grasses, the inoculation of bermudagrass with *Bacillus* sp. strains demonstrated beneficial effects, increasing the nitrogenase activity, phosphate solubilization, and siderophore production [63]. Similarly, *P*. *megaterium* exhibited plant defense and growth-promoting responses in *Arabidopsis thaliana* by elevating the expression of defense-related genes and increasing the accumulation of salicylic acid (SA) [64].

Another group of bacteria was the Gram-negative Pseudomonadota; they accounted for 35% of the isolated bacterial community, with nine species identified. Members of this group exhibit diverse functionalities affecting plant life. For example, *Stenotrophomonas maltophilia* (Xanthomonadales) is commonly found in agricultural environments and is known for its plant-growth-promoting characteristics [65], as well as its antifungal activity against pathogens in cereals [66]. *Pseudomonas oryzihabitans* (Pseudomonadales) is described as a soil bacterium that survives in moist, muddy environments and is indigenous to rice paddies [67].

### 4.4. Bacterial Endophytes Across Grass Growth Types and Habitats

Diverse bacterial communities have been reported in the roots of grasses across many species in different habitats [15,30,60,68,69]. In our study, the highest bacterial diversity was obtained in the open-grassland *F*. *arundinacea*, with nine species (18 isolates) recovered, followed by *L*. *temulentum*, with seven species (12 isolates). Among the factors influencing more bacterial vs. fungal endophyte hosting could be the dry soil conditions in an open-field grassland compared to the damp habitat established under the forest canopy, the niche of the woodland *F*. *gigantea*. In addition, certain plant species may be specifically favorable for hosting diverse endophytes and potentially benefit from interactions that positively affect their growth and stress tolerance. The importance of plant host specificity to the fungal endophyte abundance in the forage grasses *Phleum pratense* and *L*. *perenne* was demonstrated by Przemieniecki et al. [27]. Our results from the roots align with their findings from the leaves, showing that *L*. *perenne* has a particularly low endophyte occurrence among that of other grasses used in agricultural grasslands.

Interestingly, the lack of a correlation between the microbial communities of the *L*. *perenne* × *F*. *gigantea* hybrids and their parental species suggests that endophyte recruitment is influenced by complex host–microbe interactions rather than simple genetic inheritance. Quantitively, these laboratory-produced hybrids did not show an enhanced presence of microbial endophyte taxa. On the other hand, the species of natural hybrid origin, *F*. *arundinacea* and *F*. *gigantea*, harbored greater microbial diversity compared to that of their diploid *F*. *pratensis* and *L*. *perenne* relatives, showing a possible link between hybrid-derived genome complexity and increased root microbiota.

A network analysis of microbial communities identified Burkholderiales as consistently present across diverse ecosystem types and as a keystone taxon in grasslands, forests, and agricultural lands [70]. We identified two representatives of Burkholderiales in the grass roots: *Achronobacter spanius* and the *Variovorax* sp. Moreover, our analysis detected *Pedobacter alluvionis*, a member of Sphingobacteriales that, according to Banerjee and co-authors [66], is also among the keystone taxa in woodland and grassland ecosystems. Therefore, although the diversity of the endophytes identified in this study is relatively limited compared to that of the meta-analysis data, our findings confirm the presence of key microbial community members within the roots of *Festuca* and *Lolium* grasses.

Our results are based on a location in Lithuania that features common grassland in the European temperate climate zone. The impact of location on endophytic microbial diversification is significant. For instance, a study of the bacterial and fungal endophytes in *Elymus nutans* (Poaceae) seeds revealed that the fungal community in the seeds varied significantly across four locations on the Qinghai–Tibet Plateau, whereas the bacterial community was not affected by the plant’s growth location [71]. Further investigation into *Festuca* and *Lolium* species in different locations and environmental conditions (e.g., drought, cold, and contrasting soils) would provide a deeper understanding of the core endophyte microbiome transmitted across generations and its modulation by environmental factors.

## 5. Conclusions

Our study reveals distinct patterns of fungal and bacterial endophyte diversity in *Festuca* and *Lolium* grasses, with variations linked to the host species, growth type traits, and ecological adaptations. The annual species *L*. *multiflorum* and *L*. *temulentum* exhibited a greater abundance of endophytes in the roots than that of their perennial relatives, possibly reflecting a need for broader symbiotic interactions to support their short life cycles. In the woodland grass *F*. *gigantea*, higher fungal endophyte diversity was observed compared to that in the open-grassland *Festuca* and *Lolium* perennials. This implies that environmental factors such as light and humidity, along with access to organic matter, are key players in shaping microbial communities. Differences in the microbial colonization and diversity across *Festuca* and *Lolium* grasses—between perennials and annuals as well as between forest-adapted and open-field species—provide valuable insights into the ecological roles of endophytes. Overall, our data from a grassland site in Lithuania suggest that both environmental and genetic factors influence endophyte colonization. Further investigations across contrasting environments and locations are needed to gain a deeper understanding of the core endophyte microbiome of these species and its modulation by environmental factors. Among the root-derived endophytes isolated, several fungi and bacteria are potential candidates for plant growth promotion and biocontrol. These isolates include the fungi *Aureobasidium pullulans*, *Cadophora fastigiata*, *Epicoccum nigrum*, and *Plectosphaerella cucumerina*, as well as the bacteria *Priestia megaterium* and *Stenotrophomonas maltophilia*.

## Figures and Tables

**Table 1:** List of plant species assessed in the current study.

Species	Ploidy Level	Growth Type	Habitat Type	Accession Origin
*Festuca arundinacea* ‘Monas’	2n = 6x = 42	Perennial	Open-grassland	LCAFS, IA
*Festuca gigantea*	2n = 6x = 42	Perennial	Forest sites	VU BG, Kairėnai, Vilnius, LT; Vingis Park, Vilnius, LT
*Festuca pratensis* ‘Alanta’	2n = 2x = 14	Perennial	Open-grassland	LCAFS, IA
*Lolium perenne* ‘Veja’	2n = 2x = 14	Perennial	Open-grassland	LCAFS, IA
*Lolium multiflorum* ‘Grazer’	2n = 2x = 14	Annual	Open-grassland	LCAFS, IA
*Lolium temulentum*	2n = 2x = 14	Annual	Open-grassland	IPK Leibniz Institute, Gatersleben, DE
*Lolium perenne* × *Festuca gigantea*	2n = 4x = 28	Perennial	Open-grassland	Laboratory-produced, VU BG laboratory collection

Note: LCAFS, IA—Lithuanian Centre for Agricultural and Forestry Sciences, Institute of Agriculture, Akademija, Kėdainiai distr., LT; VU BG—Botanical Garden, Vilnius University; n—the haploid chromosome number: 2n—the total chromosome number in an organism; x—the basic chromosome number: 2x—diploid species, 4x—tetraploid species, 6x—hexaploid species.

**Table 2:** The list of primers in the PCR reactions for the amplification of fungal and bacterial DNA sequences.

Locus	Primers	Primer Sequences (5′–3′)	Tm °C	Reference
**Primers for fungal DNA**				
ITS	ITS1 ITS4	TCCGTAGGTGAACCTGCGG TCCTCCGCTTATTGATATGC	54	[34]
*TEFa*	EF1-278F EF-2	CATCGAGAAGTTCGAGAAGG GGARGTACCAGTSATCATGTT	54	[29]
*SSU*	NS1 NS4	GTAGTCATATGCTTGTCTC CTTCCGTCAATTCCTTTAAG	49	[34]
*RPB2*	RPB2-5F2 fRPB2-7cR	GGGGWGAYCAGAAGAAGGC CCCATRGCTTGYTTRCCCAT	58	[35]
**Primers for bacterial DNA**				
16S rDNA	27f CM 1492R	AGAGTTTGATCMTGGCTCAG TACGGYTACCTTGTTACGACTT	52	[28]
16S rDNA	704F 765R	GTAGCGGTGAAATGCGTAGA CTGTTTGCTCCCCACGCTTTC	56	[28]
16S rDNA	S-D-Bact-0341-b-S-17 S-D-Bact-0785-a-A-21	CCTACGGGNGGCWGCAG GACTACHVGGGTATCTAATCC	56	[36]

**Table 3:** Endophytic fungal and bacterial taxon richness (D) across different plant species. * No.—number; ** D—Margalef’s index.

Plant Species	Endophytic Fungi			Endophytic Bacteria			Total Microbial Endophyte Abundance	
	* No. Isolates	No. Species	** D	No. Isolates	No. Species	D	No. Isolates	No. Species
**Perennial species**								
*Festuca arundinacea*	1	1	1.00	18	9	2.77	19	10
*Festuca gigantea*	7	6	2.57	7	7	3.08	14	13
*Festuca pratensis*	1	1	1.00	8	7	2.89	9	8
*Lolium perenne*	2	2	1.44	3	3	1.82	5	5
*Lolium perenne* × *Festuca gigantea*	7	4	1.54	5	4	1.86	12	8
**Annual species**								
*Lolium multiflorum*	27	9	2.43	6	4	1.67	33	13
*Lolium temulentum*	15	5	1.48	12	7	2.41	27	12

## Data Availability

All of the data are contained within the article.

## Supplementary Materials

The following supporting information can be downloaded at https://www.mdpi.com/article/10.3390/microorganisms13040799/s1. Figure S1: Cytomorphological images; Table S1: Taxonomic assignment of the endophytic fungi; Table S2: Isolation frequency (IF); Table S3: Taxonomic assignment of the endophytic bacteria; Table S4: The 16S DNA sequences of the bacterial isolates deposited into GenBank.
