# Supplementary material for: Identifying Root-Associated Endophytic Fungi and Bacteria in Festuca and Lolium Grasses from a Site in Lithuania
Source: Microorganisms. 2025 Mar 31;13(4):799. doi: 10.3390/microorganisms13040799 (PMC12029494; doi:10.3390/microorganisms13040799)
Supplement: Supplementary file 1 [file microorganisms-13-00799-s001.zip › microorganisms-3526171-supplementary/priedai/Figure S1_Cytomorphological images.pdf]

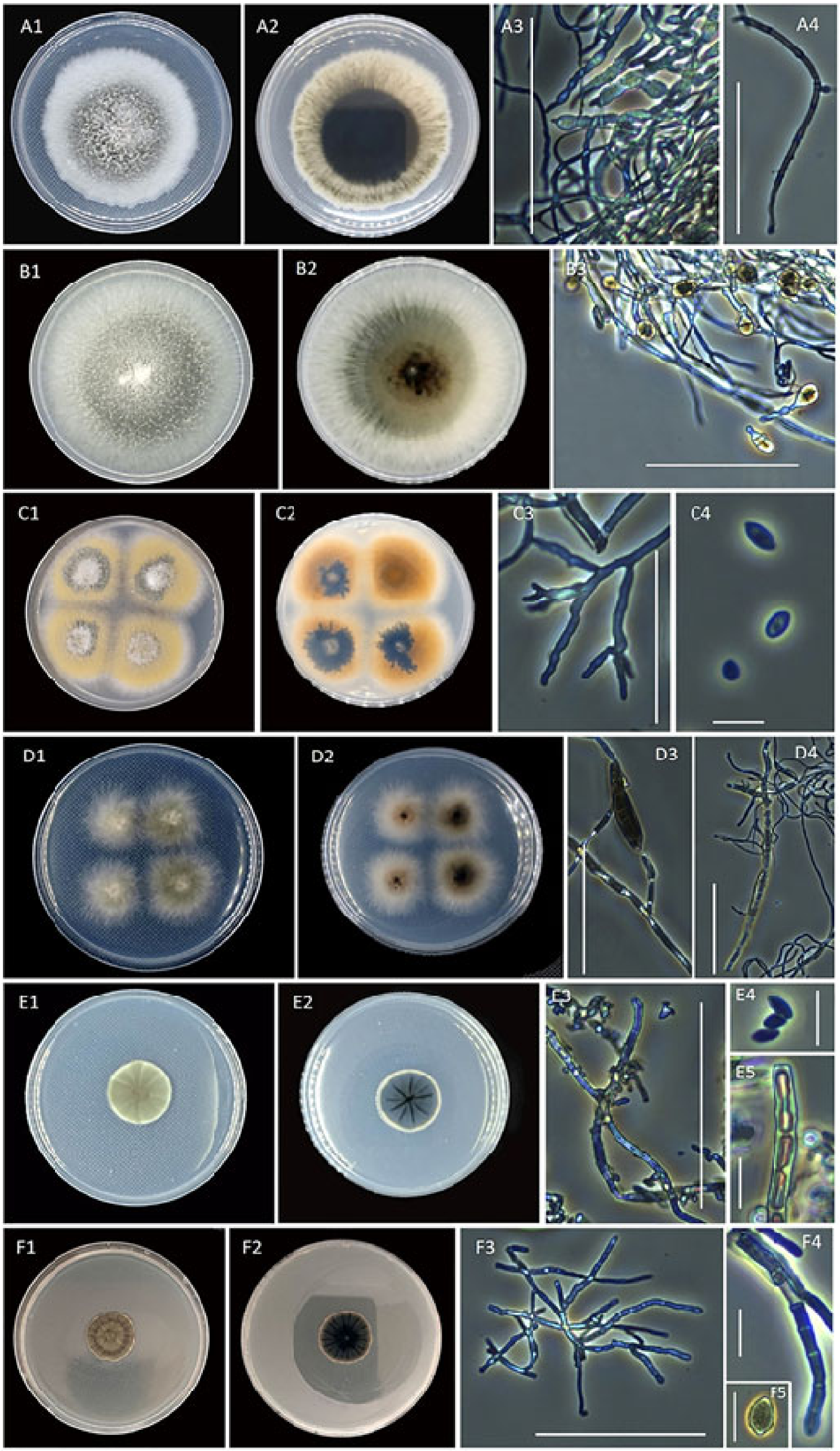
A1
A2
A3
A4
B1
B2
B3
C1
C2
C3
C4
D1
D2
D3
D4
E1
E2
E3
E4
E5
F1
F2
F3
F4
F5

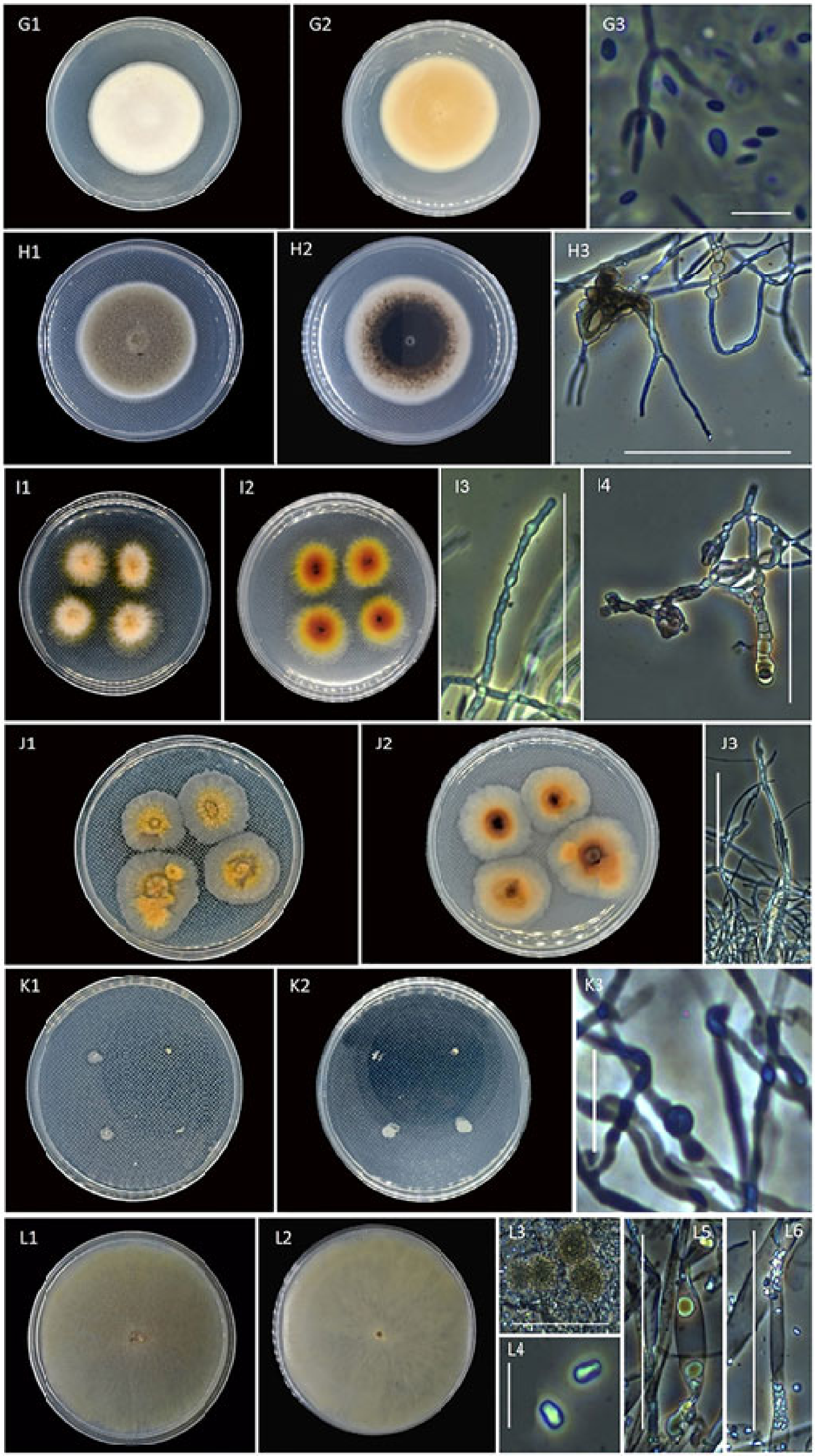
G1
G2
G3
H1
H2
H3
I1
I2
I3
I4
J1
J2
J3
K1
K2
K3
L1
L2
L3
L4
L5
L6

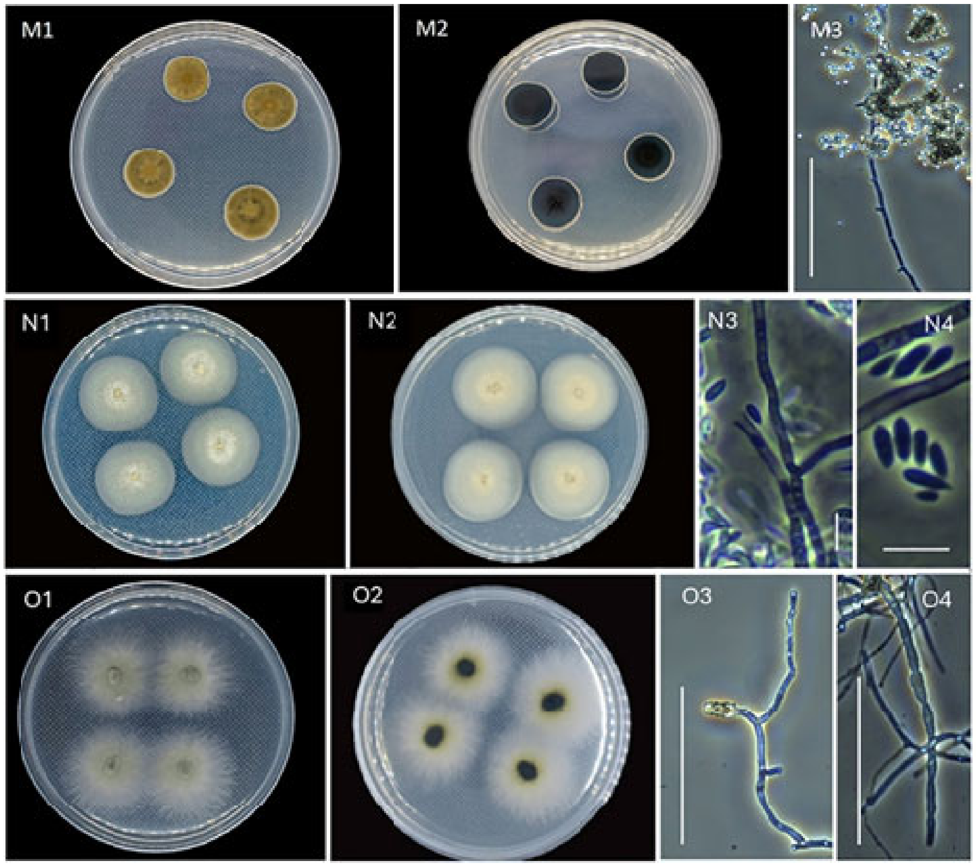

Figure S1. The 7–10-day isolate colonies on PDA medium (top image on the left, reverse on the right) from the roots of *Festuca* spp., *Lolium* spp. and in their hybrids; cytomorphological images obtained from mycelium as follows: (**A1**,**A2**) *Alternaria infectoria* (isolate BSG0013), (**A3**) conidia and conidiophores, (**A4**) segmented hyphae; (**B1**,**B2**) *Alternaria rosae* (BSG052), (**B3**) conidia with segmented conidiophores and hyphae; (**C1**,**C2**) *Aureobasidium pullulans* (BSG059), (**C3**) hyphae, (**C4**) conidia; (**D1**,**D2**) *Bipolaris sorokiniana* (BSG021), (**D3**) conidiophores, (**D4**) hyphae; (**E1**,**E2**) *Cladosporium cladosporioides* (BSG037), (**E3**,**E5**) conidiogenous cell, (**E4**) conidia; (**F1**,**F2**) *Cladosporium halotolerans* (BSG0014), (**F3**,**F4**) segmented hyphae, (**F5**) conidia; (**G1**,**G2**) *Cordyceps fumosorosea* (BSG015), (**G3**) conidiophore and conidia; (**H1**,**H2**) *Didymella macrostoma* (BSG023), (**H3**) chlamydospores; (**I1**,**I2**) *Epicoccum nigrum* (BSG025), (**I3**) pseudohyphae, (**I4**) blastoconidia and chlamydospores; (**J1**,**J2**) *Hypoxylon rubiginosum* (BSG009), (**J3**) hyphae; (**K1**,**K2**) *Lomentospora* sp. (BSG0012), (**K3**) hyphae and conidia; (**L1**,**L2**) *Mucor circinelloides* (BSG030), (**L3**) sporangia, (**L4**,**L5**) sporangiospores, (**L6**) hyphae containing lipid bodies; (**M1**,**M2**) *Paraphoma fimeti* (BSG010), (**M3**) conidiogenous cell and conidia; (**N1**,**N2**) *Plectosphaerella cucumerina* (BSG006), (**N3**) hyphae and conidia, (**N4**) conidia; (**O1**,**O2**) *Pyrenophora dictyoides* (BSG031), (**O3**,**O4**) conidia and conidiophore. Scale bar: (**A3**, **A4**, **B3**, **C3**, **D3**, **D4**, **E3**, **F3**, **H3**, **I3**, **I4**, **J3**, **L5**, **L6**, **M3**, **O3**, **O4**) = 100 μm; (**C4**, **E4**, **E5**, **F4**, **F5**, **G3**, **K3**, **L3**, **L4**, **N3**, **N4**) = 10 μm. Note: All the species are classified under Ascomycota, except for *Mucor circinelloides* (**L1**-**L6**) from Mucoromycota. The fungal morphotypes of the remaining six species that are detected in *F. gigantea* can be found in [25].
