# Supplementary material for: Identifying Root-Associated Endophytic Fungi and Bacteria in Festuca and Lolium Grasses from a Site in Lithuania
Source: Microorganisms. 2025 Mar 31;13(4):799. doi: 10.3390/microorganisms13040799 (PMC12029494; doi:10.3390/microorganisms13040799)
Supplement: Supplementary file 1 [file microorganisms-13-00799-s001.zip › microorganisms-3526171-supplementary/priedai/Table S1_Taxonomic assignment of endophytic fungi isolated.pdf]

Table S1. Taxonomic assignment of the endophytic fungi isolated from the roots of *Festuca* spp., *Lolium* spp., and *Lolium perenne* × *Festuca gigantea* according to the standard DNA data.

Fungus	Isolate Code	DNA Locus	DNA Identities, bp	Congruence, %	BLAST ID
Alternaria alternata	BSG001	ITS	440/440	100.00	PP218262.1
	BSG002		453/453	100.00	MN615420.1
	BSG016		578/578	100.00	MK649895.1
	BSG017		559/559	100.00	MN622992.1
	BSG018		390/390	100.00	PP911430.1
	BSG019		486/486	100.00	MN249500.1
	BSG020		487/487	100.00	MT644140.1
	BSG048		486/486	100.00	MT646481.1
	BSG051		520/521	99.81	MT487774.1
			670/672	99.70	OR226484.1
			522/523	99.81	OR662184.1
			671/673	99.70	LC749799.1
			522/522	100.00	MT487769.1
			672/673	99.85	AY154682.1
			522/523	99.81	MN826221.1
			654/656	99.70	MW017709.1
			522/523	99.81	PP499324.1
			670/672	99.70	KX090314.1
		*RPB*2	916/916	100.00	MK684150.1
			587/587	100.00	MN922279.1
			427/427	100.00	MG873562.1
			448/448	100.00	PP351921.1
			515/516	99.81	MT043354.1
			509/510	99.80	MT043353.1
			912/913	99.89	MN649031.1
			913/913	100.00	MN615843.1
			1041/1043	99.81	MK757628.1
		SSU	647/647	100.00	OR453387.1
			607/607	100.00	PP292027.1
			617/617	100.00	OR366485.1
			581/581	100.00	MN072922.1
			592/592	100.00	OR366486.1
			1051/1054	99.72	ON055699.1
			829/829	100.00	OM630609.1
		TEF	373/373	100.00	MK386655.1
Alternaria infectoria	BSG013	ITS	442/442	100.00	MT635276.1
	BSG058		523/523	100.00	MN560094.1
	BSG063		533/533	100.00	MN534845.1
		SSU	953/953	100.00	U43465.1
Alternaria rosae	BSG052	ITS	537/537	100.00	LR134073.1
	BSG050		554/554	100.00	MW288704.1
			521/521	100.00	LR134076.1
Aureobasidium pullulans	BSG059	ITS	528/528	100.00	MH931262.1
	BSG061		533/533	100.00	MN922107.1
	BSG071		852/853	99.88	XM_029907989.1
	BSG072		495/495	100.00	MG812620.1
	BSG073	*RPB*2	438/438	100.00	PP920516.1
		SSU	542/542	100.00	KY294714.1
			851/851	100.00	KM388547.1
Bipolaris sorokiniana	BSG021	ITS	480/481	99.79	MH538292.1
	BSG022		642/642	100.00	MN097070.1
		*RPB*2	650/650	100.00	HF934839.1
			552/552	100.00	MH014993.1
		SSU	464/464	100.00	KM111205.1
Cadophora fastigiata	BSG003	ITS	531/531	100.00	MN833359.1
			508/508	100.00	MF077223.1

Fungus	Isolate Code	DNA Locus	DNA Identities, bp	Congruence, %	BLAST ID
Chaetomium funicola	BSG039	ITS	633/633	100.00	PP165499.1
			248/249	99.60	FN394680
		SSU	417/417	100.00	AF048794.1
Cladosporium cladosporioides	BSG037	ITS	508/508	100.00	MW255614.1
			654/654	100.00	MF472941.1
		SSU	1058/1061	99.72	OR243761.1
Cladosporium halotolerans	BSG014	ITS	444/445	99.78	MN859971.1
	BSG065		448/448	100.00	MF473116.1
			493/494	99.80	MF473108.1
		SSU	586/586	100.00	MT000326.1
		TEF	476/476	100.00	MF473519.1
Coprinellus disseminatus	BSG004	ITS	464/471	98.51	FN386275
			528/535	98.69	MK077856.1
			691/700	98.71	MZ493094.1
			390/397	98.24	JN689938
Cordyceps fumosorosea	BSG015	ITS	652/652	100.00	OY756928.1
	BSG066		542/543	99.82	OY756922.1
	BSG067		430/430	100.00	MH532834.1
	BSG068		491/491	100.00	ON796011.1
	BSG069		609/609	100.00	OR758802.1
			532/533	99.81	OR758801.1
			452/453	99.78	OR121546.1
			600/600	100.00	MF872372.1
			503/503	100.00	MT333241.1
		SSU	492/492	100.00	MH879640.1
			429/429	100.00	MH879638.1
			505/505	100.00	MW391722.1
			411/411	100.00	OR194098.1
			595/595	100.00	MN576761.1
		TEF	385/385	100.00	OY756920.1
Didymella macrostoma	BSG023	*SSU*	489/489	100.00	MT649577.1
			632/632	100.00	KX519725.1
Epicoccum nigrum	BSG025	ITS	457/457	100.00	MT557339.1
		*RPB*2	475/475	100.00	PP333174.1
		SSU	432/432	100.00	MF072589.1
Hypoxylon rubiginosum	BSG009	ITS	516/516	100.00	MH319948.1
			604/604	100.00	OQ831968.1
			575/575	100.00	MW907965.1
Lomentospora sp.	BSG012	ITS	626/710	88.17	MT316371.1
		SSU	1048/1058	99.05	U43910.1
Microdochium bolleyi	BSG007	ITS	460/460	100.00	MT276137.1
	BSG008		458/458	100.00	MT276118.1
	BSG011		553/553	100.00	OL898490.1
	BSG024		469/469	100.00	MT446207.1
	BSG026		448/449	99.78	MT446123.1
	BSG027		466/466	100.00	MT446104.1
	BSG028		466/466	100.00	MT446101.1
	BSG029		457/457	100.00	KP859018.1
	BSG036		499/501	99.60	MT102452.1
	BSG054		590/590	100.00	KY305060.1
	BSG055		500/500	100.00	OM647846.1
	BSG064		499/500	99.80	OM743901.1
		*RPB*2	574/576	99.65	MN313335.1
			543/543	100.00	MN817764.1
			611/612	99.84	OP184898.1
			523/523	100.00	OP184897.1
			666/668	99.70	MN817751.1
			503/505	99.60	MK212911.1

Fungus	Isolate Code	DNA Locus	DNA Identities, bp	Congruence, %	BLAST ID
		SSU	810/810	100.00	MN817764.1
			867/870	99.66	OP184897.1
			720/721	99.86	HM216190.1
			835/836	99.88	HM216190.1
Mucor circinelloides	BSG030	ITS	503/505	99.60	MT603934.1
		SSU	518/518	100.00	KM527227.1
Paraphoma fimeti	BSG010	ITS	640/649	98.61	AB488489.1
			710/710	100.00	KF251675.1
			1236/1252	98.72	MF494612.1
Plectosphaerella cucumerina	BSG006	ITS	426/426	100.00	MT447472.1
	BSG062		453/453	100.00	MZ206376.1
	BSG070		573/573	100.00	KC427067.1
			455/456	99.78	LR594792.1
		*RPB*2	544/544	100.00	GU180612.1
		SSU	522/524	99.62	ON935484.1
Pyrenophora dictyoides	BSG031	ITS	517/518	99.81	MK044599.1
	BSG035		502/505	99.41	JN943653.1
	BSG049		515/518	99.42	MT192329.1
	BSG053		526/530	99.25	MH859312.1
	BSG056		665/670	99.25	MK539981.1
	BSG057		1021/1021	100.00	JN940962.1
		SSU	576/576	100.00	JN940958.1
Sistotrema brinkmannii	BSG005	ITS	514/514	100.00	DQ093653.1
			650/650	100.00	JQ912675.1
			548/548	100.00	MT159987.1
			567/567	100.00	KM222227.1
		SSU	556/556	100.00	R464262.1
