# Supplementary material for: Identifying Root-Associated Endophytic Fungi and Bacteria in Festuca and Lolium Grasses from a Site in Lithuania
Source: Microorganisms. 2025 Mar 31;13(4):799. doi: 10.3390/microorganisms13040799 (PMC12029494; doi:10.3390/microorganisms13040799)
Supplement: Supplementary file 1 [file microorganisms-13-00799-s001.zip › microorganisms-3526171-supplementary/priedai/Table S2_Isolation frequency (IF).pdf]

Table S2. Isolation frequency (IF) of endophytic microorganisms isolated from the roots of *Festuca* spp., *Lolium* spp. and *Lolium perenne* × *Festuca gigantea*.

No.	Endophytic fungal species	Isolation frequency (IF), %	Endophytic bacteria genus	Isolation frequency (IF), %
1.	*Microdochium bolleyi*	20.00	*Bacillus*[Bc]	37.29
2.	*Alternaria alternata*	15.00	*Priestia*[Bc]	13.56
3.	*Pyrenophora dictyoides*	10.00	*Kosakonia*[P]	8.47
4.	*Aureobasidium pallulans*	8.33	*Paenibacillus*[Bc]	6.78
5.	*Cordyceps fumosorosea*	8.33	*Pseudomonas*[P]	6.78
6.	*Alternaria infectoria*	5.00	*Actinoallomurus*[A]	3.39
7.	*Plectosphaerella cucumerina*	5.00	*Pantoea*[P]	3.39
8.	*Alternaria rosea*	3.33	*Peribacillus*[Bc]	3.39
9.	*Bipolaris sorokiniana*	3.33	*Achromobacter*[P]	1.69
10.	*Cladosporium halotolerans*	3.33	*Heyndrickxia*[Bc]	1.69
11.	*Cadophora fastigiata*	1.67	*Lysinibacillus*[Bc]	1.69
12.	*Chaetomium funicola*	1.67	*Niallia*[Bc]	1.69
13.	*Cladosporium cladosporioides*	1.67	*Novosphingobium*[P]	1.69
14.	*Coprinellus* sp.[B]	1.67	*Pedobacter*[Bt]	1.69
15.	*Didymella macrostoma*	1.67	*Robertmurraya*[Bc]	1.69
16.	*Epicoccum nigrum*	1.67	*Sphingomonas*[P]	1.69
17.	*Hypoxylon rubiginosum*	1.67	*Stenotrophomonas*[P]	1.69
18.	*Lomentospora* sp.	1.67	*Variovorax*[P]	1.69
19.	*Mucor circinelloides*[M]	1.67		
20.	*Paraphoma fimeti*	1.67		
21.	*Sistotrema brinkmannii*[B]	1.67		

All fungi belong to Ascomycota, except for *Coprinellus* sp. and *Sistotrema brinkmannii* from Basidiomycota (indicated as B) and *Mucor circinelloides* from Mucoromycota (indicated as M).

The bacterial phyla are indicated as follows: Bacillota (Bc) – (N=8); Pseudomonadota (P) – (N=8); Actinomycetota (A) – (N=1); Bacteroidota (Bt) – (N=1).
