# Supplementary material for: Identifying Root-Associated Endophytic Fungi and Bacteria in Festuca and Lolium Grasses from a Site in Lithuania
Source: Microorganisms. 2025 Mar 31;13(4):799. doi: 10.3390/microorganisms13040799 (PMC12029494; doi:10.3390/microorganisms13040799)
Supplement: Supplementary file 1 [file microorganisms-13-00799-s001.zip › microorganisms-3526171-supplementary/priedai/Table S3_Taxonomic assignment of endophytic bacteria isolated.pdf]

Table S3. Taxonomic assignment of the endophytic bacteria isolated from the roots of *Festuca* spp., *Lolium* spp., and *Lolium perenne* × *Festuca gigantea* according to standard 16S rDNA sequences.

**Bacteria**	**Colony Characteristics**		**DNA Identities, bp**	**Congruence, %**	**BLAST ID**
	**Colony Shape, Surface, Edge Shape**	**Color**			
Achromobacter spanius	Round, slightly raised, edge smooth	White, light brown	1077/1079	99.81	KP860309.1
Actinoallomurus sp.	Almost round, matte, very raised, uneven edge	Gray white	1082/1085	99.72	AB604841.1
			1089/1090	99.91	LC325912.1
Bacillus cereus	Feathery, non-protruding, roughened (granulated), edge smooth	Gray white	1083/1084	99.91	MH130346.1
			1084/1084	100.00	OP986940.1
Bacillus licheniformis	Round and irregular shape, rough and wrinkled, opaque, uneven edge	White	1078/1078	100.00	MH305323.1
			1094/1094	100.00	KP418813.1
			1083/1086	99.72	MH605434.1
			1071/1074	99.72	MW487813.1
Bcillus pumilus	Almost round, opaque, shiny, uneven edge	Yellow	1088/1088	100.00	MK156165.1
			1088/1088	100.00	MK100782.1
			1090/1093	99.73	OM698827.1
			532/532	100.00	KX904728.1
			1086/1087	99.91	MK521063.1
Bacillus subtilis	Round, opaque, rough, uneven edge	White, slightly yellow	1009/1011	99.80	CP035397.1
			1080/1081	99.91	PQ061103.1
			1085/1086	99.91	MH373533.1
			1088/1089	99.91	CP031693.1
			1088/1089	99.91	MW380651.1
			1089/1091	99.82	ON878255.1
			1081/1084	99.72	MT111080.1
Bacillus sp.	Round, opaque, rough, uneven edge	White	1086/1086	100.00	MT457446.1
			1013/1044	97.03	KC236823.1
			1042/1062	98.49	PQ013662.1
			1084/1086	99.82	MT534000.1
Heyndrickxia oleronia	Round, shiny, with slightly transparent edges	Sand	1082/1087	99.54	MF662517.1
Kosakonia cowanii	Round, surface is smooth, shiny, uneven edge	White	1090/1090	100.00	PP528640.1
			1089/1090	99.91	PP528628.1
			1068/1088	98.16	MG835978.1
			1087/1087	100.00	PP528607.1
			1083/1083	100.00	PP651538.1
Lysinibacillus boronitolerans	Round, flat, opaque, smooth edge	White	1084/1087	99.72	MH385002.1
Niallia circulans	Round, rough, uneven edge	White	1083/1084	99.91	MG988220.1
Novosphingobium sp.	Round, flat, glossy, semi-transparent, smooth edge	Light brown	1097/1097	100.00	KY123877.1
			385/385	100.00	LC436024.1
Paenibacillus barengoltzii	Round, raised, opaque, smooth	Cream	1085/1086	99.91	MT509886.1
Paenibacillus sp.	Round, transparent, convex, shiny, smooth edge	White	1077/1079	99.81	MK456436.1
			1084/1084	100.00	LC769488.1
			1089/1090	99.91	CP126313.1

Pantoea agglomerans	Round, flat, shiny, smooth edge	Yellow	1086/1088<br>1091/1091	99.82<br>100.00	CP048033.1<br>ON202849.1
**Bacteria**	**Colony Characteristics**		**DNA Identities, bp**	**Congruence, %**	**BLAST ID**
	**Colony Shape, Surface, Edge Shape**	**Color**			
Pedobacter alluvionis	Round, convex, opaque, shiny, smooth edge	Pink	1067/1070	99.72	OK090501.1
Peribacillus asahii	Round, shiny, irregular edge	White	1081/1083	99.82	KY660439.1
Peribacillus frigoritolerans	Round, shiny, irregular edge	White	1086/1086	100.00	MN710450.1
Priestia aryabhattai	Round, straight edges, shiny oil, fluff-shaped colony	Cream	1084/1088<br>1081/1082	99.63<br>99.91	MH321608.1<br>MN543853.1
Priestia megaterium	Round, matte, creamy consistency, smooth, smooth edge	Whitish	532/532<br>1088/1090<br>1091/1092<br>1077/1078<br>1090/1093<br>1082/1082	100.00<br>99.82<br>99.91<br>99.91<br>99.73<br>100.00	MK263002.1<br>MF079370.1<br>MG430231.1<br>MN161199.1<br>GU323374.1<br>MT487598.1
Pseudomonas oryzihabitans	Oval, wrinkled, shiny, irregular edge	Yellow	1083/1084	99.91	PP651577.1
Pseudomonas sp.	Oval, opaque, glossy, flat, irregular edge	Yellow	1070/1072<br>1083/1084<br>1088/1088	99.81<br>99.91<br>100.00	MT102561.1<br>OR363695.1<br>MT269585.1
Robertmurraya siralis	Round, glossy, flat, smooth edge	White	1081/1081	100.00	PP542506.1
Sphingomonas sp.	Round, flat, shiny, smooth edge	Yellow	1085/1085	100.00	OR363655.1
Stenotrophomonas maltophilia	Round, raised, shiny, smooth edge	White	1090/1092	99.82	JQ579644.1
Variovorax sp.	Round, convex, edge smooth	Yellow	1081/1095	98.72	OY740253.1
