# Supplementary material for: Identifying Root-Associated Endophytic Fungi and Bacteria in Festuca and Lolium Grasses from a Site in Lithuania
Source: Microorganisms. 2025 Mar 31;13(4):799. doi: 10.3390/microorganisms13040799 (PMC12029494; doi:10.3390/microorganisms13040799)
Supplement: Supplementary file 1 [file microorganisms-13-00799-s001.zip › microorganisms-3526171-supplementary/priedai/Table S4_Bacterial GenBank ID.pdf]

Table S4. The 16S DNA sequences of the bacterial isolates have been deposited into GenBank.

Bacteria	Isolate code	GenBank ID	Sequence length (bp)
Achromobacter spanius	BSB062	PQ144546	1079
Actinoallomurus sp.	BSB001<br>BSB002	PQ144586<br>PQ144656	1085<br>1090
Bacillus cereus	BSB003<br>BSB004	PQ152314<br>PQ152315	1084<br>1084
Bacillus licheniformis	BSB005<br>BSB006<br>BSB007<br>BSB008	PQ152317<br>PQ152320<br>PQ152321<br>PQ152235	1078<br>1094<br>1086<br>1074
Bacillus pumilus	BSB020<br>BSB022<br>BSB023<br>BSB024	PQ164425<br>PQ165300<br>PQ164521<br>PQ164700	1088<br>1088<br>1093<br>532
Bacillus subtilis	BSB013<br>BSB014<br>BSB015<br>BSB016<br>BSB017<br>BSB018<br>BSB019	PQ186790<br>PQ164775<br>PQ169019<br>PQ186787<br>PQ186786<br>PQ186788<br>PQ186789	1011<br>1081<br>1086<br>1089<br>1089<br>1091<br>1084
Bacillus sp.	BSB009<br>BSB010<br>BSB011<br>BSB057	PQ147042<br>PQ148862<br>PQ148153<br>PQ151653	1086<br>1044<br>1062<br>1086
Heyndrickxia oleronia	BSB012	PQ165299	1087
Kosakonia cowanii	BSB026<br>BSB027<br>BSB028<br>BSB029<br>BSB030	PQ182342<br>PQ182363<br>PP999032<br>PQ182576<br>PQ182595	1090<br>1090<br>1088<br>1087<br>1083
Lysinibacillus boronitolerans	BSB054	PP999036	1087
Niallia circulans	BSB031	PQ182636	1084
Novosphingobium sp.	BSB060<br>BSB021	PQ198551<br>PQ198548	1097<br>385
Paenibacillus barengoltzii	BSB035	PQ199303	1086
Paenibaccilus sp.	BSB036<br>BSB039<br>BSB040	PQ203669<br>PQ198665<br>PQ203670	1079<br>1084<br>1090
Pantoea agglomerans	BSB032<br>BSB033	PQ198557<br>PQ198656	1088<br>1091
Pedobacter alluvionis	BSB034	PQ203671	1070
Peribacillus asahii	BSB050	PQ203673	1083
Peribacillus frigoritolerans	BSB038	PQ203672	1086
Priestia aryabhattai	BSB045<br>BSB056	PP999035<br>PQ203341	1088<br>1082
Priestia megaterium	BSB041<br>BSB042<br>BSB043<br>BSB044<br>BSB025	PQ203348<br>PQ203674<br>PQ203675<br>PQ203676<br>PQ208190	532<br>1090<br>1092<br>1078<br>1093

	BSB055	PQ208191	1082
Pseudomonas oryzihabitans	BSB047	PQ212749	1084
Pseudomonas sp.	BSB048<br>BSB049<br>BSB058	PQ204581<br>PQ211015<br>PQ211037	1072<br>1084<br>1088
Robertmurraya spiralis	BSB051	PQ213359	1081
Sphingomonas sp.	BSB052	PQ215938	1085
Stenotrophomonas maltophilia	BSB053	PQ216291	1092
Variovorax sp.	BSB059	PQ216293	1095
